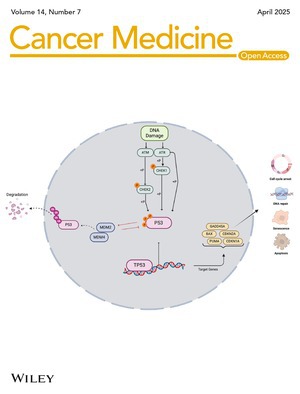# Cover Image

**DOI:** 10.1002/cam4.70874

**Published:** 2025-05-13

**Authors:** Cecilia Monge, Brigette Waldrup, Sophia Manjarrez, Francisco G. Carranza, Enrique Velazquez‐Villarreal

## Abstract

The cover image is based on the article *Detecting PI3K and TP53 Pathway Disruptions in Early‐Onset Colorectal Cancer Among Hispanic/Latino Patients* by Cecilia Monge et al., https://doi.org/10.1002/cam4.70791.